# The importance of the genomic landscape in Waldenström's Macroglobulinemia for targeted therapeutical interventions

**DOI:** 10.18632/oncotarget.16130

**Published:** 2017-03-11

**Authors:** Antonio Sacco, Adriano Fenotti, Loredana Affò, Stefano Bazzana, Domenico Russo, Marco Presta, Michele Malagola, Antonella Anastasia, Marina Motta, Christopher J. Patterson, Giuseppe Rossi, Luisa Imberti, Steven P. Treon, Irene M. Ghobrial, Aldo M. Roccaro

**Affiliations:** ^1^ ASST Spedali Civili, Coordinamento e Progettazione Ricerca Clinica, CREA Laboratory, Brescia, BS, Italy; ^2^ ASST Spedali Civili, SITRA, Brescia, BS, Italy; ^3^ ASST Spedali Civili, Collegio IPASVI, Brescia, BS, Italy; ^4^ University of Brescia Medical School, Adult Bone Marrow Transplantation Unit, Brescia, BS, Italy; ^5^ University of Brescia Medical School, Dept. of Molecular and Translational Medicine, Brescia, BS, Italy; ^6^ ASST Spedali Civili, Dept. of Hematology, Brescia, BS, Italy; ^7^ Dana-Farber Cancer Institute, Dept. Medical Oncology, Harvard Medical School, Boston, MA, USA

**Keywords:** Waldenström's Macrolobulinemia, genomics

## Abstract

The Literature has recently reported on the importance of genomics in the field of hematologic malignancies, including B-cell lymphoproliferative disorders such as Waldenström's Macrolgobulinemia (WM). Particularly, whole exome sequencing has led to the identification of the MYD88^L265P^ and CXCR4^C1013G^ somatic variants in WM, occurring in about 90% and 30% of the patients, respectively. Subsequently, functional studies have demonstrated their functional role in supporting WM pathogenesis and disease progression, both *in vitro* and *in vivo*, thus providing the pre-clinical evidences for extremely attractive targets for novel therapeutic interventions in WM. Of note, recent evidences have also approached and defined the transcriptome profiling of WM cells, revealing a signature that mirrors the somatic aberrations demonstrated within the tumor clone. A parallel research field has also reported on microRNAs (miRNAs), highlighting the oncogenic role of miRNA-155 in WM. In the present review, we focus on the latest reports on genomics and miRNAs in WM, providing an overview of the clinical relevance of the latest acquired knowledge about genomics and miRNA aberrations in WM.

## INTRODUCTION

Waldenström's Macrolgobulinemia (WM) is a B-cell malignancy, classified as a lymphoplasmacytic lymphoma, according to the WHO classification, [1] characterized by a clonal infiltration of lymphoplasma cells within the bone marrow (BM) and a serum immunoglobulin (Ig) M monoclonal component. It is considered a rare disease with an estimated incidence of around 5 per million people, over the age of 50 years old (www.rarediseases.org).

A pre-malignant condition, defined as IgM monoclonal gammopathy of undetermined significance (MGUS) may precede the WM stage disease, being characterized by less than 10% lymphoplasmacytic BM involvement, less than 3g/dL of monoclonal IgM, together with lack of clinical signs or symptoms secondary to the WM disease. Importantly, a rate of IgM MGUS-to-WM progression of about 2% per year has been reported. [2, 3] In parallel, a smoldering WM (s-WM) status may also exist, defining patients with BM lymphoplasmacytic infiltration of 10% or more, IgM monoclonal protein of 3g/dL or more, in absence of any sign or symptom of disease. Therefore, s-WM differs from IgM MGUS only for the percentage of BM involvement and the amount of circulating monoclonal IgM. In contrast, WM is characterized by the presence of more than 10% BM involvement of clonal lymphoplasma cells, monoclonal IgM of any degree, and end organ damage due to either to the circulating monoclonal component or the tumor clone itself. [3, 4] Patients may present with non-specific constitutional symptomatology, anemia or thrombocytopenia, hepato-, spleno-, lympho-adenomegalies, due to BM, liver, spleen or lymph node infiltration by tumor cells. Hyperviscosity syndrome, secondary to elevated serum IgM levels, may be responsible for blurred vision, oro-nasal bleeding or headache. 7%-20% of the patients may also present with Raynaud phenomenon, acrocyanosis, purpura and skin ulcers, due to the presence of type-I cryoglobulinemia. [5–8] The monoclonal IgM component may also display an autoimmune activity against myelin-associated glycoproteins, thus explaining the occurrence of peripheral neuropathy or against the FC portion of the IgG, thus acting as a monoclonal rheumatoid factor. [5–8] In general, tissue deposition of malignant cells will lead to signs and symptoms specific for the colonized tissue: for instance, skin nodule or macroglobulinemia *cutis* may reflect a skin involvement; while malabsorption and diarrhea or proteinuria may be due to intestine or kidney deposition of tumor cells. [5–8] In rare cases, WM cells may also infiltrate the central nervous system, leading to the so called Bing-Neel syndrome, an heterogeneous clinical condition that may be characterized by vertigo, headache, ataxia, diplopia, sensor or motor deficit, hearing problems, and possibly coma. [6, 9]

A focused area of investigation has evaluated the presence of potential copy number alterations and chromosomal translocations in WM. It has been shown that WM cells are characterized by lower genomic instability as compared to other B-cell lymphoproliferative disorders, including multiple myeloma and chronic lymphocytic leukemia: [10] for instance WM cells lack of IgH translocation, [11] rarely present with 13q14 deletions, [12] and limited karyotypic abnormalities are seen. [13] Among chromosomal abnormalities, deletions of the long arm of chromosome 6 are the most frequently observed in WM patients: [13] tumor suppressor genes mapping within this area include B lymphocyte -induced maturation protein 1 (BLIMP1) and tumor necrosis factor α-induced protein 3 (TNFAIP3). [14, 15] Lack of BLIMP1 may be part of WM pathogenesis due to its role in mediating differentiation of B cells into mature plasma cells; [15–17] while absence of TNFAIP3 may explain the constitutive activation of NFκB of WM cells, [18] due to its negative regulatory feedback on NFκB signaling activation. [19]

Several biological studies have been recently performed in the specific area of genomics, and have successfully enhanced our understanding of the molecular mechanisms underlying WM pathogenesis. The present article will provide an overview of the most recent studies in the field of genomics in WM, with a specific focus on mutational-, transcriptomal-profiling and microRNA aberrations, highlighting the most relevant findings that have led to the identification of novel target for therapies in WM.

## MUTATIONAL PROFILING IN WM CELLS AND RELATED FUNCTIONAL SEQUELAE

Dr. Treon's Group has reported for the first time on the most recurrent somatic variants in patients with WM, identified as the MYD88^L265P^ mutation, occurring in more than 90% of the patients, and being present at the IgM MGUS stage with a frequency of 50-80% of the cases. [20] These observation may lead us to hypothesize that the observed variant of the MYD88 gene may represent an early oncogenic event, present already at the IgM MGUS stage: indeed, recent evidences report on higher risk for malignant evolution towards WM. [21]

MYD88 somatic variants have been also observed in other B-cell lymphoproliferaive disorders, including chronic lymphocytic leukemia and activated B-cell-like subtype of diffuse large B-cell lymphoma (ABC-DLBCL), occurring in about 3% and 29% of the cases, respectively. [22, 23]

MYD88^L265P^ mutant has been extensively studied in ABC-DLBCLs where it exerts its oncogenic role *via* activation of pro-survival signaling pathways, including NFκB, JAK/STAT3, together with enhanced secretion of cytokines, such as IL-6, IL-10, and interferon-β. [23]

**Figure 1:**
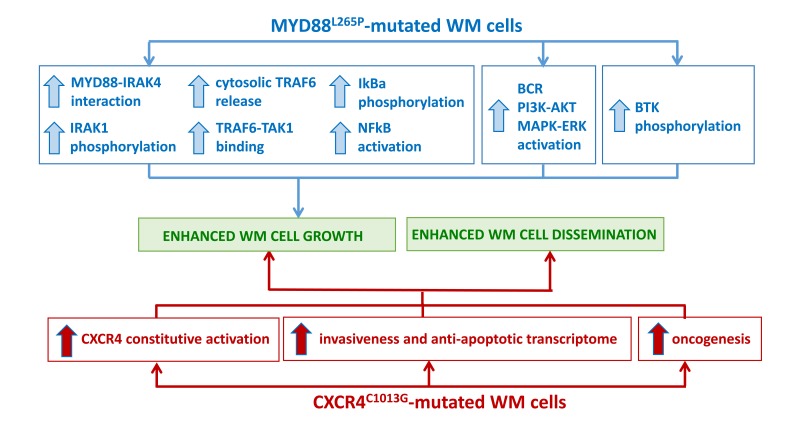
Impact of the most recurrent somatic variants on WM pathogenesis **A**. MYD88^L265P^-mutated WM cells present with enhanced NFκB activation, supported by pro-survival signaling pathway activation, responsible for WM cell growth. **B**. CXCR4^C1013G^-mutated WM cells present with constitutive CXCR4-mediated signaling, responsible for enhanced WM cell growth and tumor cell dissemination.

In the specific context of WM, the MYD88^L265P^ somatic variant may represent an early transforming event and subsequent genomic insults may lead to WM disease progression. Authors have also demonstrated the 100% occurrence of the variant in WM patients with family history, [20] findings also confirmed more recently in a separate series of studies reporting on the role of LAPTM5 and HCLS1 germline variants in WM patients with coinheritance for WM. [24] From a clinical point of view, patients who were homozygous for MYD88^L265P^ presented with a longer mean interval since the diagnosis of WM was made; no differences in terms of other clinical features and laboratory findings were observed between homozygosity and heterozygosity for this variant. [25] Authors have also characterized the functional role of this mutation and described how the MYD88^L265P^ variant confers WM cell proliferative advantage through NFκB (NFκB) activation (Figure 1A). Indeed, the use of a MYD88-homodimerization inhibitor reduced p65-NFκB activation within the nuclear compartment of MYD88^L265P^-engineered WM cells, together with decrease of IκBα, a well known NFκB inhibitor. [25] As proof of concept, Authors have further confirmed these findings demonstrating how WM cells engineered with the control construct did not present with any NFκB pathway activation, being un-responsive to MYD88 inhibition. Further studies have also revealed that MYD88 is complexed with phosphorylated Bruton tyrosine kinase (BTK) in WM cells harboring the MYD88^L265P^ variant. [25] These observations were corroborated by the demonstration that MYD88-silencing led to inhibition of BTK activation in MYD88^L265P^-engineered WM cells; while MYD88^L265P^-gain of function favored BTK phosphorylation. Importantly, the same Authors showed that the BTK inhibitor ibrutinib disrupted the MYD88-BTK interaction, leading to inhibition of MYD88^L265P^ mutated WM cell proliferation. [25] Overall, these findings have for the first time described BTK as a downstream target for the MYD88^L265P^-mutated cells, thus providing the preclinical evidence for using BTK inhibitors in WM patients harboring the MYD88^L265P^ somatic variant.

The second most recurrently mutated gene in WM is represented by C-X-C chemokine receptor type 4 (CXCR4), a G-protein coupled receptor, that acts as a key regulator of cell trafficking in hematopoietic stem cells and clonal B cells, and interacts with the related ligand stromal derived factor 1 (SDF1). [26–29] Specifically, almost 30% of WM patients present with somatic mutations of the CXCR4, one of them being the CXCR4^C1013G^ variant, [30–34] resembling what documented in patients with the WHIM (warts, hypogammaglobulinemia, infections, and myelokathexis). WHIM is a rare inherited, heterozygous, autosomal dominant disease characterized by aberrant immunity, where the CXCR4 mutations leads to truncation of the carboxy-terminal domain of the receptor, responsible for impaired CXCR4 intracellular trafficking, increased responsiveness to SDF-1 and retention of neutrophils within the BM. [35, 36]

Recent studies have characterized the functional relevance of the CXCR4^C1013G^ somatic variant, demonstrating it acts as an activating mutation, responsible for enhanced WM cell dissemination leading to disease progression *in vivo*. [34] Specifically, Authors have documented how CXCR4-mutated WM cells present with an enrichment for mRNAs related to oncogenesis, cell proliferation, tumor invasiveness and anti-apoptosis, thus further confirming the activating role of the CXCR4^C1013G^ variant in WM. Importantly, CXCR4-mutated cells also presented increased resistance to Ibrutinib treatment (Figure 1B). [32–34] These preclinical observations were also validated in the clinical setting, in WM patients enrolled in the Ibrutinib clinical trial, as described in the following sections. [37]

The third most common mutation in WM patients is represented by the AT-rich interacting domain containing protein 1A (ARID1A) gene, a chromatin remodeling protein; it occurs in the 17% of the cases. [25] ARID1A-mutated WM patients presented with elevated BM disease infiltration as compared to the non-ARID1A-mutated counterpart. [38] Of note, detection of ARID1A mutation was associated with increased level of CXCL13, known to act as modulator of mast cell recruitment. [38] This mutation may also be crucial in WM pathogenesis, considering past studies demonstrating the presence of mast cells within WM cell-infiltrated BM niches. [39]

## TRANSCRIPTOMAL CHANGES IN WM CELLS

After having identified and described the occurrence of somatic aberrations within WM cells, a recent study has also attempted, with success, to evaluate whether the mutational landscape may exert an impact at transcriptomal level. [38] In these well designed studies, Authors have performed next-generation RNA sequencing profile evaluating 57 WM patients, having healthy donor-derived B cells, as the normal cellular counterpart. [38] The results confirmed the B-cell origin of WM cells, as demonstrated by the up-regulation of variable-diversity and junctional recombination- and B-cell receptor-related genes, such as DNTT, RAG1, RAG2 and IGLL1, but not AICDA. All WM patients also presented with reduced BAX expression, high-level of BCL2. In those cases of mutated ARID1A, CXCL13 appeared to be over-expressed, showing a correlation with BM involvement and hemoglobin levels. The detection of high level of CXCL13 may open new insights into the relevance of mast cells in WM pathogenesis, since infiltration of mast cells has been described as an important and well represented component of the BM microenvironment in WM patients, as demonstrated by immunohistochemistry detection within BM tissues. [38]

Among the most significantly up-regulated mRNAs, Authors could demonstrated the presence of CXCR4, together with CXCR4 signaling-related genes, including CXCL12/SDF-1 and VCAM1, regardless of the CXCR4 mutational status, thus further confirming the crucial role of CXCR4 and CXCR4-related pathways in supporting WM disease biology. In addition to the description of the baseline transcriptional profiling in WM patients, these studies have also highlighted the importance of the mutational status in modulating gene expression. Overall, the RNA sequencing-generated data analyzed based on the CXCR4 and MYD88 mutational status provided the rational for potential stratification of patients within three different cohort: MYD88^wtild type(wt)^-CXCR4^wt^, MYD88^L265P^-CXCR4^WHIM^ and MYD88^L265P^-CXCR4^wt^, since each of them presented with a specific downstream signaling pathways. For instance, MYD88^wt^-CXCR4^wt^ patient-transcriptional landscape was characterized by reduced levels of both B-cell differentiation and NFκB responses genes, elevated expression of phosphatidylinositol 3-kinase (PI3K) signaling-related genes, and increased promoter methylation for PRDM5 and WNK2. In contrast, WM patients harboring the MYD88^L265P^-CXCR4^WHIM^ somatic variant, showed an enrichment for tumor suppressors that were, conversely, up-regulated upon MYD88 mutations; together with enhanced levels of IRAK3 and reduced expression of TLR4; or reduced levels of negative regulators of MAPK signaling pathways; or, lastly, increased expression of PIK3R5 and PIK3CG genes. In those cases with a MYD88^L265P^-CXCR4^wt^ status, the highest levels of both IGF1 and B-cell differentiation genes were demonstrated, with high level of PMAIP1, and an overall transcriptional signature that was the most distinct from healthy individuals as well as from other WM genotypes. [38]

Taken together, these represent important observations that have significantly enhanced our understanding of the molecular mechanisms that support WM pathogenesis and that will certainly have high impact in the clinical setting for WM patients.

## MICRORNA ABERRATIONS AND THEIR RELEVANCE IN WM PATHOGENESIS

The term of epigenetics has been historically defined by Sir. Conrad Waddington as “the branch of biology which studies the causal interactions between genes and their products, which bring the phenotype into being”. [40] After several years of research, epigenetics is now considered a real link between genotype and phenotype; and includes heritable changes in gene expression that occur in the absence of modifications in the nucleotide sequence, such as DNA methylation and chromatin remodeling. More recently, the Literature has indicated how non-coding RNAs may play a crucial role in modulating epigenetic events, [41] by acting together with the DNA methylation machinery and chromatin, leading to gene silencing. For instance, this is true for microRNAs (miRNAs): although miRNAs are usually not considered as conventional epigenetic markers, they can still induce an inheritable silencing effect, through cell divisions. [41]

**Figure 2:**
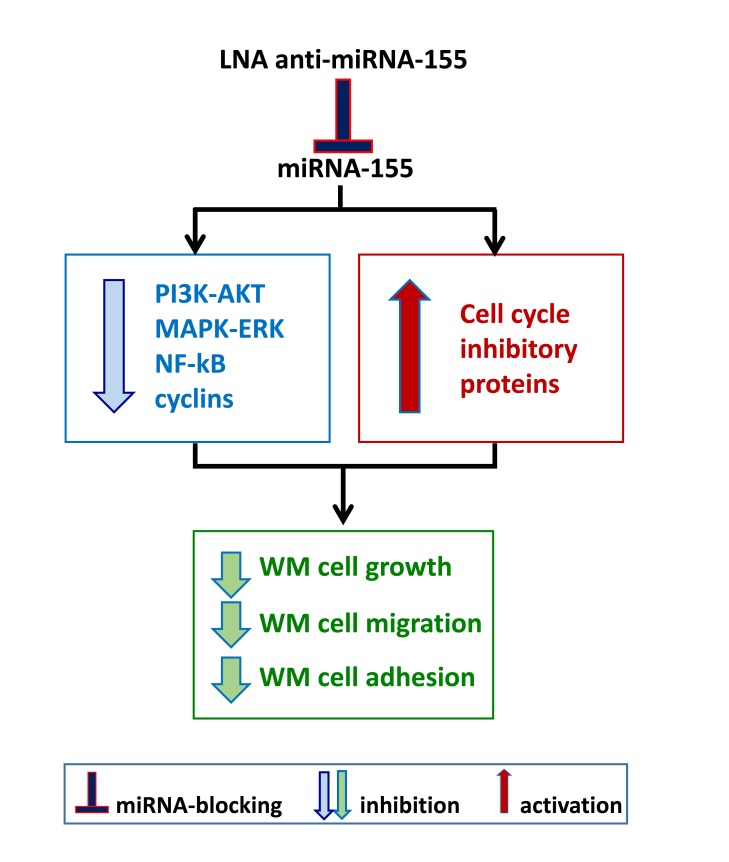
Inhibiting onco-miRNA-155 in WM cells **A**. WM cells present significantly higher levels of miRNA-155 as compared to their normal cellular counterpart. *In vitro* and *in vivo* studies have been therefore conducted in order to neutralize miRNA-155 using a lock-nucleic acid (LNA)- anti-miRNA-155, demonstrating its ability to reduce WM cell proliferation, migration and adhesion.

The first attempt to identify a potential miRNA profiling in WM patients has demonstrated the up- regulation of miRNA-155 in BM-derived WM cells as compared to their normal cellular counterpart. [42] Additional deregulated miRNAs included high levels of miRNA-184, -206, -363, -494, -542-3p, and low levels of miRNA-9. Among them, Authors have reported on the oncogenic role of miRNA-155, as demonstrated both *in vitro* and *in vivo*. [42] Specifically, miRNA-155-loss of function studies led to inhibition of WM cell proliferation supported by down-regulation of pro-survival signaling cascades, such as PI3K/AKT and MAPK/ERK; together with inhibition of NFκB activation; and reduced WM cell adhesion to fibronectin as well as inhibition of WM cell migration to SDF-1. In addition, miRNA-155-silenced cells showed reduced expression of several cyclins, including cyclin-D1, -D2, -D3, -E; and enhanced levels of cell cycle inhibitory proteins, such as cyclin-dependent kinase inhibitor-p18, -p19, -p21 and -p27. As a direct consequence, miRNA-155-silenced cells showed cell cycle modulation, with G0-G1 arrest and inhibited progression to the S-phase. [42] These *in vitro* findings were further corroborated by *in vivo* studies, demonstrating that miRNA-155-silenced WM cells presented with a reduced ability to colonize BM niches, using a disseminated WM mouse model coupled with *in vivo* confocal imaging modalities. [42] In parallel, these mice also showed prolonged survival as compared to mice injected with control probe-infected WM cells (Figure 2A).

Subsequent studies have then approached the possibility of using anti-miRNA-155 for therapeutic intervention in WM: however, the main challenge for miRNA-targeting therapies remains the ability to achieve a significant targeting of the miRNA of interest. [43] In the specific field of WM, an 8-mer lock-nucleic acid (LNA)-anti-miRNA-155 oligonucleotide with a specific target for the miRNA-155 seed region has been developed; and tested *in vivo* as a potential novel miRNA-based therapy in WM, [43] as discussed within the next section of this review.

## TRANSLATING THE ACQUIRED KNOWLEDGE IN THE FIELD OF WM GENOMIC AND MIRNA INTO THE CLINICAL SETTING: NOVEL THERAPEUTICAL INTERVENTIONS FOR WM PATIENTS

Based on preclinical findings describing the oncogenic role of either MYD88^L265P^, CXCR4^C1013G^ and miRNA-155 in WM, we now aim to provide a summary of the potential clinical impact for using agents acting against each of those targets either indirectly, as for the MYD88^L265P^ mutation, or directly, as for the CXCR4^C1013^ variant and miRNA-155.

### MYD88^L265P^ and BTK inhibition and other novel agents

The first study that has proven the anti-WM exerted by Ibrutinib in the clinical setting has evaluated 63 WM patients with relapsed-refractory disease, whose MYD88 and CXCR4 mutational status was also investigated. Treon and Collaborators reported for the first time how Ibrutinib was safe and well tolerated in patients with WM, leading to durable anti-tumor responses. [37] Specifically, an overall response rate of 90.5% was observed, with major responses occurring in 73% of the patients. Of note, patients had received a median of two lines of prior treatments, including rituximab, proteasome- and mTOR-inhibitors, immunomodulators, or traditional anti-WM chemotherapeutic. Importantly, the progression-free survival at 2 years was 69%, together with an overall survival rate of 95%. The clinical benefit was demonstrated also in the context of extramedullary WM, as documented by reduction of lymphadenopathies or splenomegaly occurring in 68% and 57% of the patients, respectively. Other extramedullary cases included 3 patients with pleural involvement and where disappearance of the pleural infiltration was shown in 2 of them. [37]

As for other agents, [44–46] such as proteasome- and mTOR-inhibitors, BTK inhibition also led to clinical responses supported by IgM monoclonal component reduction and hemoglobin level increase even in absence of any reduction of BM tumor infiltration, thus suggesting the ability of these novel agents to possibly act indirectly through an effect on the BM milieu. The 63 enrolled patients were screened for the MYD88 and CXCR4 mutational status in order to proper define the potential influence of such genomic variants in modulating responses to Ibrutinib treatment. Treon and Collaborators found that the highest rates of response were observed among patients with MYD88^L265P^/CXCR4^WT^; while a reduced response rate was demonstrated for patients with MYD88^L265P^/CXCR4^WHIM^. [37] These observations obtained within the clinical setting recapitulate what previously shown in *in vitro* studies, where WM cells engineered for the CXCR4^C1013G^ variant were characterized by a transcriptome signature enriched for drug-resistance and, specifically, were resistant to Ibrutinib treatment. [37] In terms of toxicity, moderate toxic effects were observed, including grade-2 or higher neutropenia and thrombocytopenia in 22% and 14% of the patients, respectively. Of note both neutropenia and thrombocytopenia were more common in heavily pre-treated patients. 3% of the cases showed bleeding post-procedure, and in 3% of patients who had used fish-oil supplements. Cardiotoxicity occurred in 5% of the cases, in the form of atrial fibrillation, and where all the patients had positive anamnesis for arrhythmia. [37]

A recent phase III trial, designed for treating rituximab-refractory WM patients, has further corroborated the importance for targeting BTK in WM disease. The studies showed that Ibrutinib used as single agent is significantly active and well tolerated in rituximab-refractory patients, showing an overall response rate of 90%, with major responses achieved in 71% of the cases. [47] Ibrutinib-treated patients presented with manageable toxicity, with the most frequent adverse events being either grade-1 or -2, in contrast with the occurrence of neurotoxicity, myelosuppression or pulmonary toxicity that represented major concerns and clinical challenges for existing therapies. [48–50]

Other agents that have been identified following the pre-clinical studies showing the crucial role of MYD88^L265P^ in WM, include PI3K-δ and Toll-like receptor (TLR) inhibitors. Indeed, PI3K-δ represents a transcription factor activated by MYD88^L265P^; and TLR-7, -8. -9 is also dysregulated in MYD88^L265P^ mutated WM patients. For instance, Idelalisib-dependent PI3K-δ inhibition led to an overall response rate of 70% in relapsed-refractory WM patients; while IMO-8400, an oligonucleotide antagonizing TLR-7, -8, and -9 is under investigation in clinical trials for relapsed-refractory WM patients. [7]

### CXCR4^C1013G^ and CXCR4 inhibition

Targeting CXCR4 represents another interesting therapeutic approach in patients with WM, harboring or not the CXCR4^C1013G^ mutation. Indeed, CXCR4 plays a crucial role in modulating BM homing of B-cell lymphoproliferative disorders, including also WM. [26–28, 34, 51] Specifically, the Literature has reported on the anti-WM activity of a novel antibody anti-CXCR4 (ulocuplumab) that has demonstrated to inhibit WM tumor growth both *in vitro* and *in vivo*. [34] Ulocuplumab is a fully humanized anti-CXCR4 monoclonal antibody, with half-life longer than the CXCR4 antagonist plerixafor, and that has been described as a pro-apoptotic agent in the context of both lymphoid and myeloid malignancies. [52] Ulocuplumab has been successfully used in clinical trials for the treatment for relapsed/refractory multiple myeloma (MM) and acute myeloid leukemia (AML) patients. [53, 54] A phase Ib study has used ulocuplumab in combination with either lenalidomide plus low-dose dexamethasone in patients with relapsed/refractory MM; and demonstrated ulocuplumab-dependent blockade of the CXCR4-SDF-1 axis was safe and well tolerated, inducing a high response rate in more than 50% of the patients enrolled that were already exposed to lenalidomide in previous lines of treatment. Similarly, encouraging results were obtained in patients with relapsed/refractory AML receiving ulocuplumab in combination with mitoxantrone, etoposide, cytarabine (MEC). This study showed that ulocuplumab is safe and well tolerated, leading to improved response rate obtained using MEC alone. [53]

In the specific context of WM, ulocuplumab has been recently studies both *in vitro* and *in vivo*. [34] Ulocuplumab-dependent CXCR4 neutralization was equally efficacious in targeting both CXCR4^WHIM^ mutated and un-mutated WM cells, where inhibition of phosphor(p)-AKT, p-ERK and p-SRC was documented, together with increased p-GSK3β and p-β-catenin, leading to β-catenin degradation. [34] Importantly, ulocuplumab was able to also exert a direct anti-WM activity as demonstrated by induction of caspase-9 and PARP cleavage. [34] Overall, these observations provide the preclinical rational for testing ulocuplumab, a fully humanized anti-CXCR4 monoclonal antibody for the treatment of WM patients, harboring or not the CXCR4^WHIM^ mutation, in the context of clinical trials.

### Onco-miRNA-155 and locked-nucleic-acid (LNA)-anti-miRNA-155 inhibition

It is widely accepted the concept that miRNA-dependent regulation of gene expression is of pivotal importance in supporting tumor pathogenesis and this has been described both in solid tumors as well as in hematologic malignancies. [55, 56] Within the specific area of B-cell lymphoproliferative disorders, miRNA-155 exerts an oncogenic role in several conditions, including chronic lymphocytic leukemia, diffuse large B-cell and primary mediastinal B-cell lymphomas. [57–59] In addition, transgenic mice generated by over-expressing miRNA-155 in B cells were characterized by pre-B cell proliferation, followed by high grade B-cell lymphoma transformation. [60] More recently, we have reported on the increased miRNA-155 levels in BM-derived WM cells, as compared to their normal cellular counterpart obtained from healthy donors. [42] Overall, these findings have provided the evidence for categorizing miRNA-155 as an onco-miRNA; and for considering miRNA-155 a novel potential target for therapy in B-cell neoplasms. One of the main challenge for using miRNA-based therapies is to handle oligonucleotide that specifically binds to the miRNA of interest. For this reason, a class of high-affinity RNA analogs, the so called LNA, has been developed: the ribofuranose ring in the sugar-phosphate backbone of these RNA analogs is “locked” in an RNA-like, C3′-endo conformation, leading to increased affinity between LNA-modified anti-miRNA oligonucleotides and the related complementary miRNA target. [61]

Previous studies by Zhang and Collaborators have reported on the anti-WM activity of the 8-mer LNA oligonucleotides complementary to the miRNA-155 seed region, as documented in a pre-clinical setting, both *in vitro* and *in vivo*. [43] Specifically, Authors have first dissected and confirmed the delivery of a FAM-labeled anti-miRNA-155 within WM cell-infiltrated BM and splenic niches, using *in vivo* confocal microscopy: [43] this led to both inhibition of WM cell homing to the BM; and reduced WM tumor growth *in vivo*, as documented by using bioluminescence imaging modalities. Importantly, Authors defined novel targets for miRNA-155, including MAFB and CEBPβ; and confirmed *ex vivo* MAFB and CEBPβ neutralization in anti-miRNA-155-treated mice, using explanted femurs and spleen. [43] These *in vivo* findings were further corroborated by demonstrating inhibition of WM cell proliferation *in vitro*. Overall, these studies have provided the first pre-clinical evidence for testing anti-miRNA-155-based therapies in patients with WM.

## CONCLUSIONS

The last four years have enormously contributed to enhance a better understanding of the molecular mechanisms that support both WM pathogenesis and disease progression: indeed, preclinical studies, in the specific field of genomics, have demonstrated the occurrence of somatic variants of the MYD88 and CXCR4 genes, present in over 90% and 30% of the WM patients, respectively. These mutations were further dissected for their functional implications, and *in vitro* and *in vivo* studies have confirmed they act as gain-of-functions mutations, enhancing WM cell proliferation, leading to disease progression. Similarly, the onco-miRNA-155 has been also studied in WM, confirming its oncogenic role in WM.

These findings have been rapidly translated from the laboratory to the clinical setting, with the identification of novel therapeutical approaches and the initiations of several clinical trials for the treatment of patients with relapsed/refractory WM. Overall, these novel agents are demonstrating attractive anti-WM activity in the setting of WM patients with relapsed/refractory disease, thus enlarging the number of therapeutic options available for this cohort of patients, for whom only a few treatment modalities are available so far.
